# Denudation of the continental shelf between Britain and France at the glacial–interglacial timescale

**DOI:** 10.1016/j.geomorph.2013.03.030

**Published:** 2013-12-01

**Authors:** Claire L. Mellett, David M. Hodgson, Andrew J. Plater, Barbara Mauz, Ian Selby, Andreas Lang

**Affiliations:** a School of Environmental Sciences, University of Liverpool, Liverpool, L69 7ZT, UK; b The Crown Estate, 16 Burlington Place, London, W1S 2XH, UK; c School of Earth and Environment, University of Leeds, Leeds, LS2 9JT, UK

**Keywords:** Drowned landscapes, English Channel, Sea level, Straits of Dover, Continental shelf stratigraphy, Quaternary

## Abstract

The erosional morphology preserved at the sea bed in the eastern English Channel dominantly records denudation of the continental shelf by fluvial processes over multiple glacial–interglacial sea-level cycles rather than by catastrophic flooding through the Straits of Dover during the mid-Quaternary. Here, through the integration of multibeam bathymetry and shallow sub-bottom 2D seismic reflection profiles calibrated with vibrocore records, the first stratigraphic model of erosion and deposition on the eastern English Channel continental shelf is presented. Published Optical Stimulated Luminescence (OSL) and ^14^C ages were used to chronometrically constrain the stratigraphy and allow correlation of the continental shelf record with major climatic/sea-level periods. Five major erosion surfaces overlain by discrete sediment packages have been identified. The continental shelf in the eastern English Channel preserves a record of processes operating from Marine Isotope Stage (MIS) 6 to MIS 1. Planar and channelised erosion surfaces were formed by fluvial incision during lowstands or relative sea-level fall. The depth and lateral extent of incision was partly conditioned by underlying geology (rock type and tectonic structure), climatic conditions and changes in water and sediment discharge coupled to ice sheet dynamics and the drainage configuration of major rivers in Northwest Europe. Evidence for major erosion during or prior to MIS 6 is preserved. Fluvial sediments of MIS 2 age were identified within the Northern Palaeovalley, providing insights into the scale of erosion by normal fluvial regimes. Seismic and sedimentary facies indicate that deposition predominantly occurred during transgression when accommodation was created in palaeovalleys to allow discrete sediment bodies to form. Sediment reworking over multiple sea-level cycles (Saalian–Eemian–early Weichselian) by fluvial, coastal and marine processes created a multi-lateral, multi-storey succession of palaeovalley-fills that are preserved as a strath terrace. The data presented here reveal a composite erosional and depositional record that has undergone a high degree of reworking over multiple sea-level cycles leading to the preferential preservation of sediments associated with the most recent glacial–interglacial period.

## Highlights

• We resolve continental shelf stratigraphy by integrating geophysics with core data.
• Chronometric data links shelf processes to major climate/sea-level periods.
• Palaeogeography of the eastern English Channel from MIS 6 to MIS 1 is reconstructed
• The timing and mechanism of erosion and deposition on the shelf is examined.
• We present evidence of noncatastrophic erosion in the English Channel.

## 1 Introduction

Significant changes in relative sea level repeatedly submerge and expose shallow continental shelves making them susceptible to erosion, reworking and deposition, by sedimentary processes operating in terrestrial, marine, and transitional environments. Consequently, the preservation of ancient landscapes on the sea bed of the continental shelf provides archives of palaeoenvironmental change (Fedje and Josenhans, 2000; Fitch et al., 2005; Gaffney et al., 2007; Kelley et al., 2010; Hijma et al., 2012), commonly from time periods poorly represented on land (Mellett et al., 2012a). These drowned landscapes are ideal for examining the interactions between sedimentary processes over glacial–interglacial sea-level cycles, and the factors that determine the imprint they leave (both erosional and depositional) on the continental shelf. The number of detailed case studies is growing rapidly due to recent advances in submarine technologies and the increasing availability of commercially acquired data, thus greatly improving our understanding of submarine landscape evolution. These advances are providing crucial evidence for assessing the impacts of future sea-level rise on coastal economies and ecosystems.

The continental shelf between Britain and France has received attention in the literature for many years (see Gibbard and Lautridou, 2003; Preece, 1995 for reviews, and Dingwall, 1975; Kellaway et al., 1975; Roep et al., 1975; Smith, 1985; Gibbard et al., 1988; Smith, 1989; Gibbard, 2007; Gupta et al., 2007; Toucanne et al., 2009a) because of its potential to preserve a record of the timing and mechanism that led to the isolation of Britain, as a geographical island, from the European continent during the mid-Quaternary. Despite ongoing debates regarding the timing of erosion (Meijer and Preece, 1995; van Vliet-Lanoë et al., 2000; Busschers et al., 2008; Hijma et al., 2012), the most recent evidence supports a Marine Isotope Stage (MIS) 12 age, at least for the initial breach (Toucanne et al., 2009a), with an English Channel–North Sea marine connection during highstand, at some point between MIS 12 to MIS 6 (Meijer and Cleveringa, 2009). The resulting palaeogeographical configuration of Britain and Northwest Europe has implications for the migration of flora and fauna (Preece, 1995; Meijer and Cleveringa, 2009) including hominids (Stringer, 2006; Hijma et al., 2012) throughout the Pleistocene. Additionally, reorganisation of drainage basins and funnelling of freshwater discharge through the English Channel during cold stages as a consequence of breaching of the Straits of Dover (Gibbard et al., 1988; Bridgland et al., 1993; Bridgland and D'Olier, 1995; Gibbard, 1995; Busschers et al., 2007, 2008), may have contributed to destabilisation of the Atlantic thermohaline circulation (Menot et al., 2006; Gibbard, 2007; Toucanne et al., 2009b, 2010).

A number of mechanisms have been proposed to explain breaching at the Straits of Dover including gradual erosion as a result of fluvial downcutting (Dingwall, 1975; Gibbard et al., 1988; Busschers et al., 2008) and catastrophic flooding (Smith, 1985; Gibbard, 2007; Gupta et al., 2007). The bedrock morphology of the continental shelf in the eastern English Channel, particularly the erosional bedrock bedforms preserved in the Northern Palaeovalley, have been interpreted as the product of high magnitude flows linked to erosion at the Straits of Dover by megaflood events (Gupta et al., 2007). However, this interpretation was based on bathymetric data of the sea bed only, and did not consider the sedimentary record preserved in the subsurface stratigraphy. Distinguishing catastrophic events from ‘normal’ sedimentary processes requires an understanding of how fluvial and marine processes, over multiple sea-level cycles, interact to create the morphological and sedimentary history of the continental shelf.

Here, the first detailed chronometrically constrained stratigraphic record of palaeoenvironmental change on the eastern English Channel continental shelf is presented. The erosional and depositional record is established through the integration of multi-beam bathymetric data with subsurface geophysical and vibrocore data. Tying sedimentary and morphological data to available chronometry (Wessex Archaeology, 2008; Mellett et al., 2012b) permits reconstructions of palaeogeographic- and drainage configurations. Catastrophic flood events are discussed in the light of new insights into the timing and nature of erosion and deposition on the continental shelf.

## 2 Geographical and geological setting

**Fig. 1:**
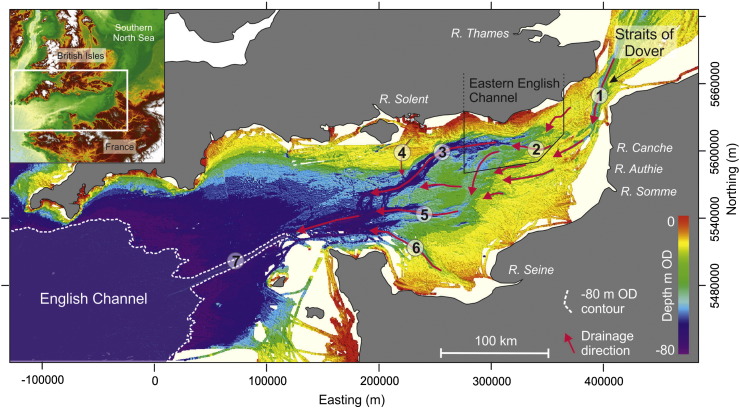
Sea bed bathymetry of the English Channel continental shelf. Inset map shows merged bathymetric and topographic data for Northwest Europe (Bathymetry: The GEBCO_08 Grid, version 20091120, http://www.gebco.net. Digital elevation data: SRTM (Jarvis et al., 2008) available from http://srtm.csi.cgiar.org). Bathymetry source for the main map is Olex, used with permission of Olex AS. Depths are relative to UK mean sea-level (OD). Arrows indicate major drainage configurations and numbers identify offshore extensions of large European rivers and main palaeovalleys/topographic features: (1) Lobourg Channel, (2) South Basserelle (SB) Palaeovalley, (3) Northern Palaeovalley, (4) Palaeo-Solent, (5) Median Palaeovalley (Antoine et al., 2003), (6) Palaeo-Seine, (7) Hurd Deep. Coordinate system WGS84 UTM Zn 31 N.

The English Channel, or La Manche, is a narrow seaway that during sea-level highstands separates the terrestrial environments of southern Britain and northern France. The narrowest and shallowest tract is at the Straits of Dover in the East, and the widest and deepest tract is between Cornwall and Brittany in the West. The continental shelf submerged beneath the English Channel forms a gently sloping platform (ca. 0.01°) that extends 600 km westwards from the Straits of Dover towards the continental shelf edge break in water depths of − 200 m Ordnance Datum (OD) (Pantin and Evans, 1984). In the north-east, this slope is dissected by a complicated network of palaeovalleys that locally form offshore extensions of contemporary rivers such as the Seine, Somme and Solent (Lericolais, 1997; Velegrakis et al., 1999; Bridgland, 2002; Antoine et al., 2003; Lericolais et al., 2003; Tessier et al., 2010) (Fig. 1). These palaeovalleys are tributaries of a major axial fluvial system (Channel River/Fleuve Manche) that channelled discharge from the Thames and Rhine–Meuse rivers (Gibbard et al., 1988; Lericolais, 1997) along with meltwater from Northwest European Ice Masses (Toucanne et al., 2009b, 2010) during late Quaternary sea-level lowstands (Fig. 1). Large palaeovalleys of the Channel River that are not linked to present day onshore drainage networks include the Lobourg Channel in the Straits of Dover, the Northern Palaeovalley in the eastern English Channel and the Median Palaeovalley in the central Channel basin (Fig. 1). The course of the Channel River to the shelf edge break is interrupted by the Hurd Deep, a NE–SW trending linear depression of Neogene tectonic origin that acted as a sediment sink when the shelf was exposed during sea-level lowstands (Lericolais et al., 1996).

**Fig. 2:**
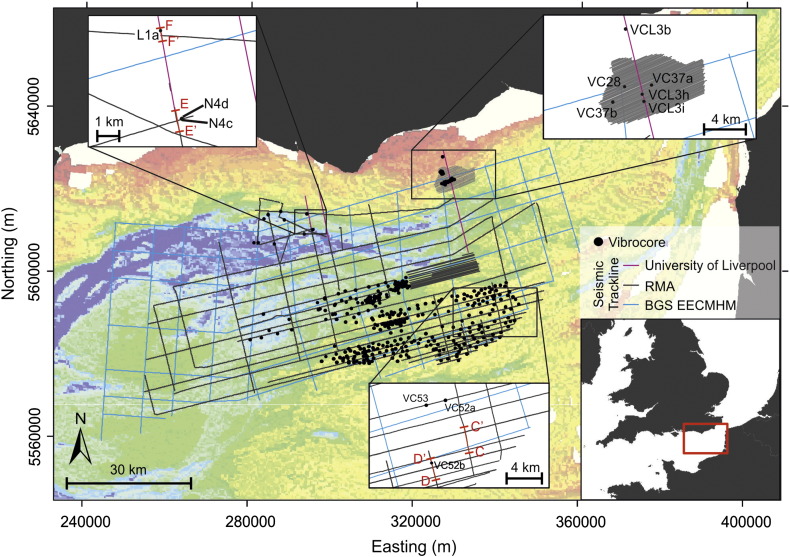
Offshore seismic and vibrocore data. Bathymetric data were extracted from the Olex database, used with permission of Olex AS. Enlarged inset maps show the location of seismic profiles presented in Figs. 4–6, and the positioning of vibrocores referred to in Figs. 10 and 11, and Table 3. Map coordinates WGS84 UTM Zn 31 N.

The area referred to in this paper as the eastern English Channel represents ca. 5000 km^2^ of the sea bed offshore of the south-east coast of Britain (Fig. 1). The extent of this study area is delimited by the availability of geophysical data (Fig. 2). Sea bed morphology reveals a number of E–W to NE–SW trending confined bathymetric lows, one of which includes the Northern Palaeovalley. This morphology is the sea bed expression of a network of palaeovalleys that physically connect the Lobourg Channel to the Northern Palaeovalley and the Median Palaeovalley (Fig. 1).

The geology of the eastern English Channel is dominated by Cretaceous strata of the Weald–Artois anticline and Cenozoic deposits of the Hampshire–Dieppe Basin (Hamblin et al., 1992). A general WSW–ENE alignment of fold and fault structures is apparent. The eastern English Channel as a geological province has remained relatively stable since uplift of the Weald–Artois anticline and sedimentation in the Hampshire–Dieppe Basin in response to a late Alpine compressional phase during the Miocene (Anderton, 2000). Thus, Pliocene and Pleistocene evolution can mostly be attributed to fluvial response to long-term tectonics with higher frequency relative sea-level changes (Lagarde et al., 2003; Le Roy et al., 2011). Gradual uplift of the English Channel during the Quaternary is recorded in fluvial terrace staircases of major rivers draining southern Britain and northern France (Antoine et al., 2000; Briant et al., 2006; Westaway et al., 2006; Antoine et al., 2007) and raised beaches of the south coast of Britain (Preece et al., 1990; Bates et al., 2003; Westaway et al., 2006; Bates et al., 2010). It is likely that uplift and subsidence in response to glacio-isostasy over glacial–interglacial cycles (Lambeck, 1997; Shennan et al., 2000; Waller and Long, 2003; Busschers et al., 2008) also influenced development of the eastern English Channel.

In the eastern English Channel study area up to 70% of the sea bed is characterised as bedrock exposed at the sea bed or bedrock covered by a thin (< 1 m) veneer of sediment (James et al., 2011). Significant thicknesses (up to 30 m) of sediment are limited to infilled portions of palaeovalleys and a small number of constructional bedforms (Hamblin et al., 1992). Sediments are predominately siliciclastic, comprising sand and gravel. Fine grained and organic-rich sediments are rare and limited to drowned palaeovalleys (Bellamy, 1995; Velegrakis et al., 1999; Gupta et al., 2004) or back barrier-settings (Mellett et al., 2012a), proximal to the present-day coastline.

## 3 Methods

### 3.1 Bathymetric data collection and processing

To determine the depth to sea bed relative to Ordnance Datum (OD), i.e. the British reference altitude of mean sea level at Newlyn, a range of bathymetric data sources were used, depending on the resolution required. Single-beam and multi-beam echo soundings were collected simultaneously with seismic reflection data (see Section 3.2 and Fig. 2 for survey track lines). The bathymetric data are used to calibrate sea bed response from seismic reflection profiles to OD. SeaZone Solutions Ltd. digital bathymetry and digital charted bathymetry were obtained and interpolated using a standard kriging algorithm in ArcGIS into a 120 m cell size grid. This allowed the morphology of the sea bed to be assessed and provided a local surface to which all other interpolations could be tied. The Olex global bathymetry database (www.olex.no) was used to characterise regional bathymetry outside the study area (Fig. 1). These data were collected using standard single-beam echo sounders and GPSs. Data coverage in the English Channel is exceptional producing a high resolution chart (between 20 m and 200 m track spacing) of the sea bed. Positioning accuracy is generally < 10 m and vertical resolution in water depths of < 100 m is 0.1 m (Bradwell et al., 2008).

### 3.2 Seismic data acquisition and interpretation

Shallow sub-bottom, 2D seismic reflection data were collected over 20 years of prospecting surveys by the Resource Management Association (RMA, comprising CEMEX UK Marine Ltd., Hanson Aggregates Marine Ltd. and Tarmac Marine Dredging Ltd.), and made available for this study. These seismic data were collected using surface-towed Boomer sources typically operating at frequencies between 0.5 and 5 kHz that penetrate and resolve unconsolidated sediments to ca. 50 m below the sea bed. These data were collated with 2D seismic reflection data collected as part of the Eastern English Channel Marine Habitat Map Project (James et al., 2007). Additional shallow sub-bottom seismic reflection data (Boomer 1–4 kHz) were collected as part of this study to permit integration and correlation between individual surveys and improve spatial resolution where data coverage was sparse. Through the integration of all datasets a total of 6000-line km of seismic reflection data were used to assess the nature, extent and thickness of sediments preserved in the eastern English Channel. The locations of all survey data are presented on Fig. 2.

**Fig. 3:**
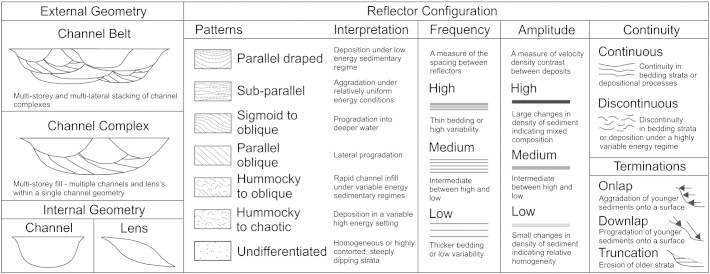
Guide to the interpretation of seismic reflection data based on Mitchum et al. (1977).

**Fig. 4:**
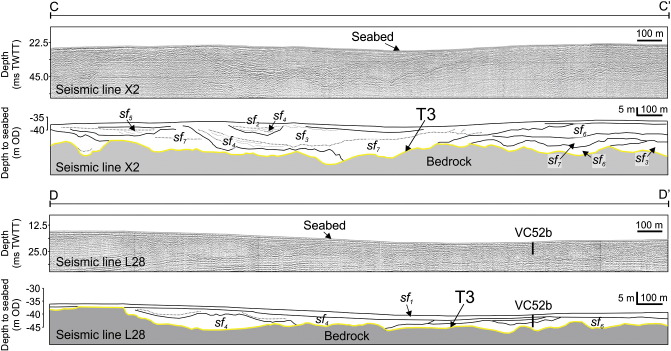
Seismic reflection profile and interpreted panels illustrating seismic facies character and association for deposits overlying surface T3. The location of profiles C–C' and D–D' are highlighted on Fig. 2. A sedimentary log of VC52b is shown in Fig. 11.

**Fig. 5:**
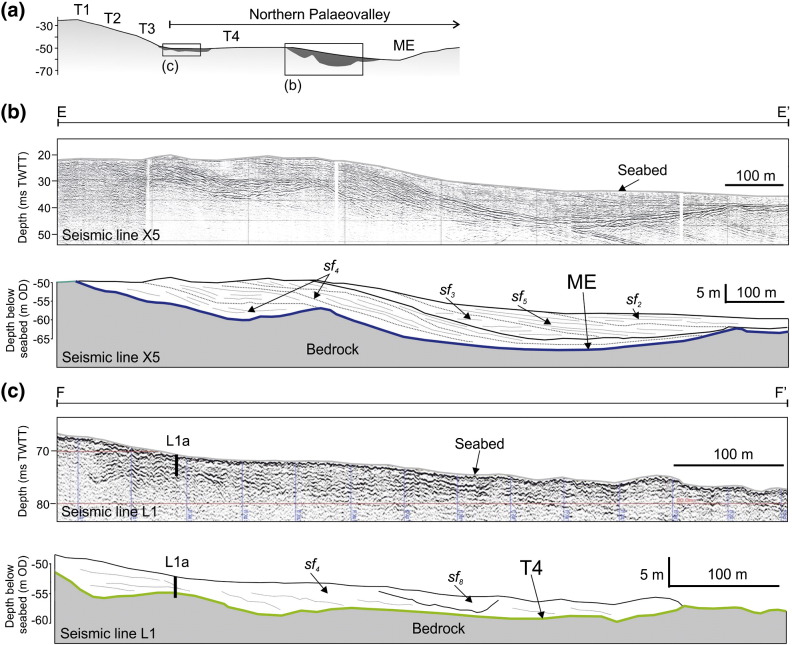
Seismic reflection profile and interpreted panels illustrating seismic facies character and association for deposits overlying surface T4 and ME. (a) Cross-profile showing bathymetry of the sea bed and the location of deposits overlying surfaces T4 and ME. (b) Seismic reflection profile and interpreted panels illustrating seismic facies character and association for deposits overlying surface ME. The location of profile E–E' is highlighted on Fig. 2. (c) Seismic reflection profile and interpreted panels illustrating seismic facies character and association for deposits overlying surface T4. The location of profile F–F' is highlighted on Fig. 2. A sedimentary log of vibrocore L1a is presented in Fig. 11.

**Fig. 6:**
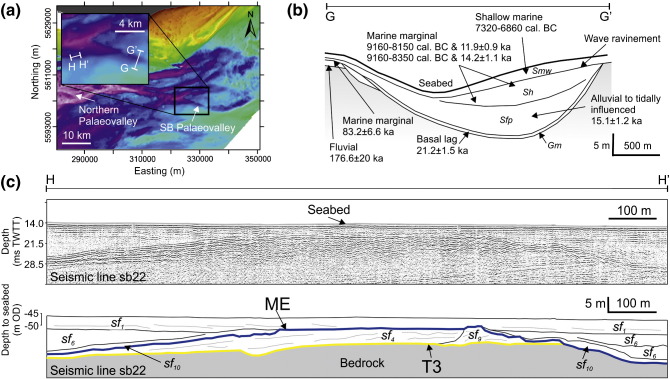
Seismic reflection profile and interpreted panels illustrating seismic facies character and association for deposits overlying surface T3 and ME. (a) Bathymetry in the eastern English Channel showing the morphology of the SB Palaeovalley at its confluent with the Northern Palaeovalley (Bathymetry © British Crown and SeaZone Solutions Limited. All rights reserved. Product license No. 112010.009). Bathymetry is shaded according to the scale given in Fig. 7a. Locations of cross-profiles G–G' and H–H' are shown as white lines. (b) Schematic representation showing the stratigraphy and depositional context of sediments preserved within the SB Palaeovalley adapted from Wessex Archaeology (2008). Sedimentary facies are provided in italics. Age data obtained from samples representing each stratigraphic unit are presented. Full details of these ages are given in Table 3. OSL ages are given in ka and ^14^C ages in cal. BC. For marine marginal sediments, a single sample was extracted for OSL and ^14^C was carried out on shell material contained within that sample. (c) Seismic reflection profile and interpreted panel illustrating seismic facies character and association for deposits overlying surface T3 and ME.

Interpretation of seismic reflection profiles is based on Mitchum et al. (1977). Characterisation of seismic facies and seismic stratigraphy was carried out according to the criteria presented in Fig. 3. A summary of all seismic facies is presented in Table 1 and shown together with interpreted seismic lines in Figs. 4–6. In unconsolidated sediments, an acoustic velocity of 1700 ms^− 1^ was used to convert two way travel time (TWTT) to depth in metres.

### 3.3 Identification of erosion surfaces

**Fig. 7:**
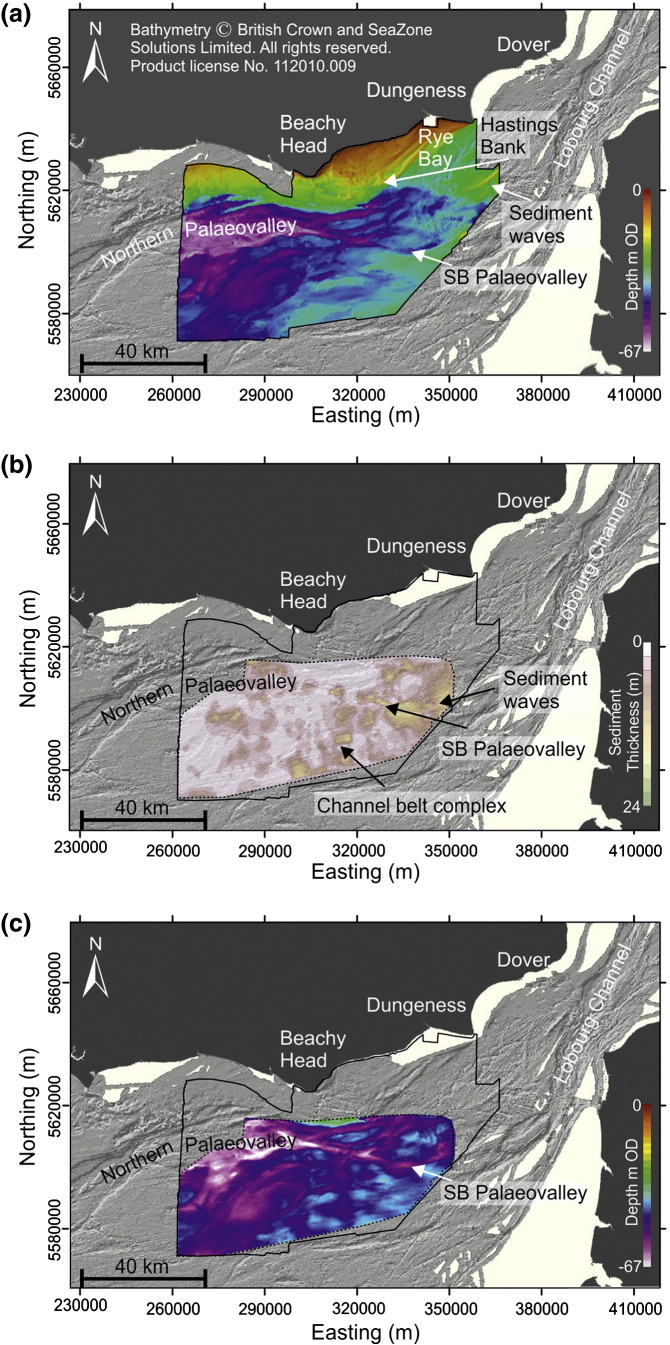
Interpolation of the unconformity separating unconsolidated sediment from bedrock in the eastern English Channel. (a) Sea bed bathymetry of the eastern English Channel study area (© British Crown and SeaZone Solutions Limited. All rights reserved. Product license No. 112010.009), overlying Olex shaded relief data, used with the permission of Olex AS. (b) Isopach of sediment thickness overlying Olex shaded relief data, used with permission of Olex AS. Solid black line delimits eastern English Channel study area. Dashed black line delimits interpolated grid. (c) Depth to bedrock shown within interpolated grid delimited by dashed black line, overlying Olex shaded relief data, used with permission of Olex AS. Map coordinates WGS84 UTM Zn 31 N.

**Fig. 8:**
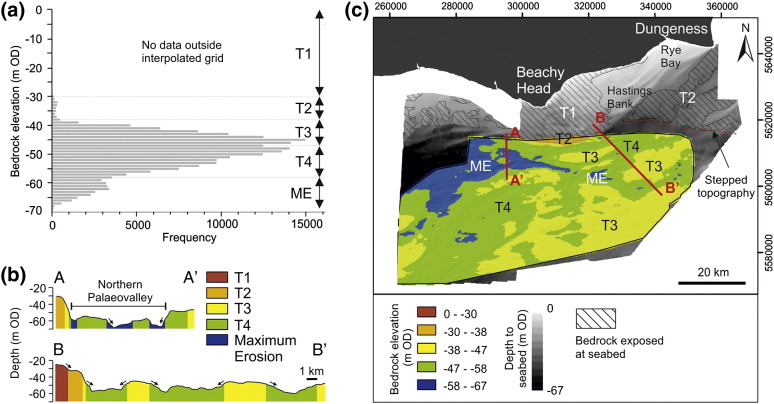
Distinction of bedrock erosion surfaces in the eastern English Channel. (a) Frequency distribution of bedrock elevations showing subdivision of erosion surfaces. (b) Cross-sections showing depth to bedrock. Locations of profiles given in Fig. 8c. Elevation of erosion surfaces shaded according to the scale in Fig. 8c. Black arrows indicate breaks in slope that distinguish different erosion surfaces. (c) Depth to bedrock shown within interpolated grid, shaded to highlight major erosion surfaces identified in Fig. 8a. Outside the interpolated grid, the depth to sea bed is given (© British Crown and SeaZone Solutions Limited. All rights reserved. Product license No. 112010.009). Areas where bedrock is exposed at the sea bed are taken from James et al. (2011). Map coordinates WGS84 UTM Zn 31 N.

**Fig. 9:**
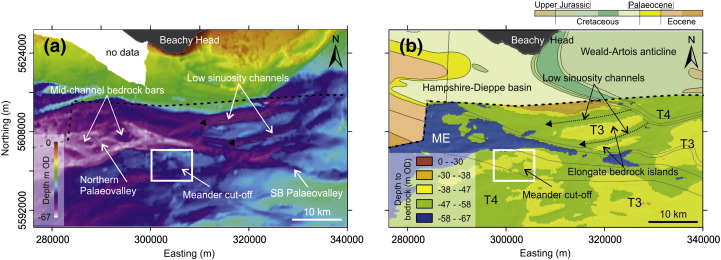
Morphology of the sea bed and bedrock erosion surface. (a) Sea bed bathymetry highlighting key morphological features (© British Crown and SeaZone Solutions Limited. All rights reserved. Product license No. 112010.009). (b) Bedrock geology (Geological Map Data © NERC 2012) overlain by an interpolated surface showing the depth to bedrock. Dashed black line delimits the extent of the interpolated grid. Map coordinates WGS84 UTM Zn 31 N.

To determine the morphology of the unconformity separating bedrock from overlying unconsolidated sediments, an isopach of sediment thickness, determined from individual seismic lines and interpolated into a 3D grid using ArcGIS (Fig. 7b), was subtracted from sea bed bathymetry (Fig. 7a) to produce a map showing bedrock elevation relative to OD (Fig. 7c). Spatial limitations of available seismic data meant this could only be carried out within a limited area referred to herein as the interpolated grid. Identification of discrete erosion surfaces within the interpolated grid was achieved by producing a frequency histogram of bedrock elevations and distinguishing between multiple populations by qualitatively identifying peaks in the distribution (Fig. 8a). To test the geomorphological significance of discrete populations associated with each peak, 2D profiles of bedrock elevation were evaluated to ensure that breaks in slope identified in the frequency histogram were true breaks in elevation separating individual erosion surfaces (Fig. 8b). Key morphological features within each erosion surface (e.g. river channels, platforms, breaks in slope and bedrock bedforms) were visually identified (Figs. 8b and 9). James et al. (2011) identify areas where bedrock is exposed at sea bed (Fig. 8c), which was used to provide additional information on the morphology of the bedrock unconformity outside the interpolated grid.

### 3.4 Vibrocoring and sedimentary facies

**Fig. 10:**
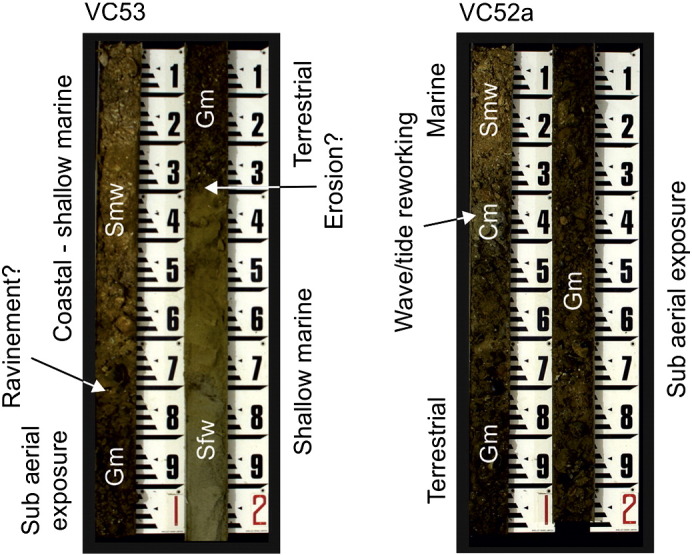
Photographs showing the composition of sedimentary facies in two vibrocores taken from the locations shown in Fig. 2. Each core comprises two sections that are 1 m in length.

**Fig. 11:**
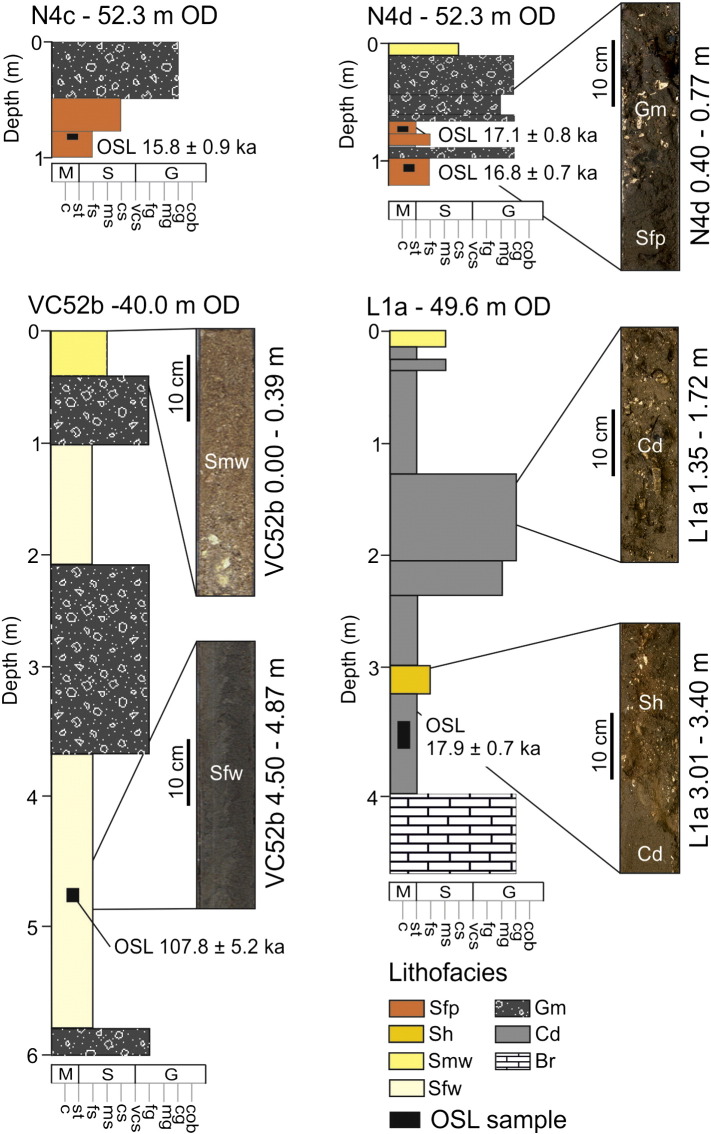
Sedimentary logs and core photographs showing stratigraphy and lithological composition of sedimentary facies discussed in Table 2. Locations of vibrocores are highlighted in Fig. 2 and correlated to seismic lines in Figs. 4 and 5. The location of OSL samples and resulting ages (Mellett et al., 2012b) are given.

A database containing over 300 vibrocores tied to seismic reflection profiles was made available by the RMA. This database included core descriptions logged in accordance with BS5930 (1981), core photographs and the results of particle size distribution analysis carried out at the discretion of the RMA within each core. All cores were recovered with a high-powered vibrocorer (6 m maximum penetration) over a series of individual surveys that corresponded with the collection of seismic reflection data. Sub-samples of these cores were selected to allow higher resolution sedimentary facies descriptions and sampling for lithological and chronometric analyses. An additional 11 vibrocores were collected during March 2010 to supplement the core database, to calibrate seismic facies with sedimentary facies, and to obtain samples for chronometric analysis (Mellett et al., 2012b). The locations of vibrocores are indicated on Fig. 2. Data obtained from the core database and vibrocore samples form the basis for lithostratigraphic analysis and characterisation of sedimentary facies. A summary of all sedimentary facies is presented in Table 2 and representative core photographs are shown in Figs. 10 and 11.

### 3.5 Chronometric data

A total of 9 optical stimulated luminescence (OSL) ages were obtained using sand-sized quartz extracted from a selection of vibrocores, full methodological details and results are published in Mellett et al. (2012b). This chronological information was supplemented by published OSL and radiocarbon (^14^C) ages from sediments deposited in the South Basserelle (SB) Palaeovalley (Fig. 6b) (Wessex Archaeology, 2008). A summary of all ages including information pertaining to the accuracy of published ages is presented in Table 3.

## 4 Results

Peak analysis of the frequency distribution of bedrock elevations revealed four major erosion surfaces referred to as T2, T3, T4 and maximum (deepest) erosion (ME) surface (− 67 m OD) (Fig. 8a). An additional erosion surface (T1) is characterised according to sea bed elevations outside of the interpolated grid where bedrock is exposed at sea bed (Fig. 8c). A framework for establishing the relative stratigraphy of erosion surfaces assumes denudation through time in an area of overall tectonic uplift (Davis, 1899). This implies surface T1 is the oldest surface at the highest elevations and ME is the youngest base level of the most recent erosional regime prior to submergence of the continental shelf by post-glacial sea-level rise. Erosion surfaces and depositional facies are discussed in chronological order from oldest to youngest.

### 4.1 Bedrock erosion

Erosion surface T1 lies outside of the interpolated grid and occupies elevations between 0 m and − 30 m OD (Fig. 8c). The erosion surface dips at 0.1° towards the south and gently westward (0.02°) towards the continental shelf break (Fig. 8c).

Erosion surface T2 lies within the interpolated grid at elevations between − 30 m OD and − 38 m OD. Outside of the interpolated grid, bedrock is exposed at sea bed at these elevations implying surface T2 is present to the NE of the interpolated grid where it appears as a planar topographic platform (Fig. 8c).

Surface T3 is the most widespread surface within the interpolated grid and it displays a strong degree of continuity at elevations between − 38 m and − 47 m. As a whole it is characterised by a relatively planar gently dipping (ca. 0.05° westwards) continuous platform (Fig. 8c). Cut into this surface is a network of cross-cutting bedrock channels with planform geometries ranging from low sinuosity linear channels to a meander cut-off indicative of higher degrees of sinuosity (Fig. 9). Surface T3 only occurs on strata of the Hampshire–Dieppe Basin. The low angle but well defined slope (0.5° to 3.0°) that separates this surface from surfaces T1 and T2 has been defined as the margin of the Northern Palaeovalley (Smith, 1985, 1989; Hamblin et al., 1992; Antoine et al., 2003; Gupta et al., 2007).

Surface T4 occupies elevations between − 47 m and − 58 m, and in the NE forms the base of WSW–ENE trending, sub-parallel, channels (on average 15 km length and 1.5 km width) that subdivide surface T3 into elongate bedrock islands (Fig. 9b). To the SW, surface T4 broadens into a 20 km-wide depression (Fig. 9) that is part of a large SW–NE trending palaeovalley that extends westwards towards the Hurd Deep (Fig. 1).

The base of the maximum erosion (ME) surface is planar at − 67 m OD. It is incised into surface T4 and creates a confined channel (the SB Palaeovalley Fig. 9a) that widens towards the west to form the base of the Northern Palaeovalley (Fig. 9a). The SB Palaeovalley follows structural folds in the bedrock geology, incising into and running parallel with London Clay at its contact with underlying chalk until it reaches Eocene-aged stratigraphy exposed in the base of the Northern Palaeovalley. Within the Northern Palaeovalley, isolated remnants of T4 form a collection of streamlined mid-channel bedrock islands (Fig. 9a).

### 4.2 Depositional facies and environments

#### 4.2.1 Deposits on surfaces T1 and T2

Bedrock is exposed at the sea bed across large areas of surface T1 (Fig. 8c). The thickest sediment cover is observed in the northeast around Hastings Bank and Rye Bay (Fig. 8c). Depositional architecture and geomorphic change in the Hastings Bank area are addressed in Mellett et al. (2012a). In summary, a seaward-thickening wedge has been identified comprising sand overlain by coarse grained gravel ridges that are breached by finer grained silty deposits. The entire succession has been interpreted as a submerged barrier complex of Holocene age. Other deposits in the Rye Bay have been characterised as a seaward prograding shelf sand body with a minor coarse clastic component (Dix et al., 1998). Elsewhere on surface T1, sediment is confined to the fills of palaeovalleys that drained the southern British uplands (Bellamy, 1995; Gupta et al., 2004).

Deposition on surface T2 is limited to sandy sediment waves and banks (Fig. 7a). These relict sediment banks are inactive under the present-day hydrodynamic regime (Anthony, 2002) but probably represent deposition during the mid- to late Holocene sea-level rise when tidal currents were influential at these depths (Uehara et al., 2006).

#### 4.2.2 Deposits on surface T3

Surface T3 is the most extensive surface in the eastern English Channel making a simple classification of associated deposits difficult. The most volumetrically significant sedimentary deposits of the eastern English Channel are associated with this surface and form a sheet of sediment that thins to bedrock in the west and is truncated by erosion surfaces T4 and ME in the north and west (Fig. 7b). Deposits overlying T3 have a composite and complicated seismic signature comprising multiple discontinuous erosion surfaces overlain by oblique to chaotic reflector patterns (Fig. 4). This seismic facies association is interpreted to represent a multi-storey and multi-lateral amalgamated channel belt (Miall, 2006) (Fig. 3). The channel belt extends 30 km to the west and 20 km to the north within the area delimited as the interpolated grid and is on average less than 6 m thick (Fig. 7b). Channel incision is either directly into bedrock or through alluvium until the bedrock is reached. The characteristic seismic stratigraphy and depositional architecture of individual palaeovalleys within the channel belt are shown in Fig. 4, and full descriptions of seismic and sedimentary facies are given in Tables 1 and 2.

Identifying single channels can be achieved on N–S trending seismic profiles where channels are intersected at 90° to their longitudinal profile and can be traced downslope across seismic profiles (Fig. 4). Channel margins cut into alluvium with channel thalweg erosion into bedrock. Asymmetric cross-sections of the channel bodies combined with oblique to sigmoid reflector patterns (sf_4_) at the channel margins indicate some degree of sinuosity, which is also reflected in the morphology of surface T3 (Fig. 9). Assuming channels are intersected perpendicular to flow direction, individual channels are on average between 0.5 and 1.5 km wide.

Sedimentary infill is complicated, with a number of smaller cut-and-fill cycles confined within the composite channel geometry. Oblique downlapping seismic reflectors (Sf_4_) are limited to the steepest margin of the channels suggesting deposition is driven by lateral- and downstream-accretion of bar complexes (Fig. 4). Discontinuous, hummocky to chaotic reflectors of Sf_7_ (Table 1) indicative of contrasting lithologies are present at the base of the channel implying that deposition occurred under a variable process regime. Sub-parallel reflectors of Sf_3_ (Table 1) representing lateral continuity in lithology, correspond to the latest stages of sedimentary infill and indicate aggradation under a more consistent energy regime.

Precise calibration of sedimentary facies with seismic facies is problematic due to the resolution of both seismic and core records that are biased towards superficial strata and thus the later stages of channel infill. Typically, cores reveal gravels supported by a sand matrix (Gm) interbedded with fine grained well-sorted, laminated sands (Sfw) (Table 2 and Fig. 10). Coarse-grained, flint-rich gravels are most likely the product of local bedrock erosion and later transport by fluvial processes within the channel belt complex. The abundance of shallow marine foraminifera (predominantly *Elphidium* sp. and *Ammonia* sp.) in Sfw indicates deposition in a shoreface setting and these sedimentary facies are considered the product of flooded valley infilling during sea-level rise. The intercalation of fluvial and shallow marine environments is reflected in the amount of reworked thick-walled shells within the coarse grained deposits. Distinguishing between marine reworked fluvial deposits and fluvial reworked marine deposits is challenging but implies deposition occurred in an environment where the position of the shoreline fluctuated in response to sea-level oscillations. In summary, deposits overlying surface T3 are interpreted as a composite sheet of sand and gravel that represent widespread interfingering and reworking by marine and fluvial processes.

Thinning of deposits overlying surface T3 to the west is attributed to an erosional event as seen from the truncation of seismic reflectors and deposition of sf_1_ (Table 1 and Fig. 4) which is evident on seismic profiles throughout the eastern English Channel. The erosion surface at the base of Sf_1_ is interpreted to represent wave ravinement during transgression due to its spatial extent and the planar nature of the lower bounding surface. Parallel onlapping seismic reflectors and the uniform thickness of sedimentary facies Smw and Cm (Table 2 and Fig. 10) suggest deposition subsequently occurred in a marine environment.

One of the most striking features of the channel belt deposits is variations in colour within sedimentary facies Gm (Table 2 and Fig. 10). The gravels are clearly heavily weathered and show evidence, based on their colour, of different degrees of secondary iron oxide development (Hurst, 1977). This would suggest that subsequent to deposition, channel belt sediments were sub-aerially exposed for a sufficient period to develop a red to dark brown coloured soil prior to erosion by wave ravinement.

#### 4.2.3 Deposits on surfaces T4 and ME

Sediments associated with surface T4 occur in isolated patches and are usually smaller than the spatial resolution of the available data. Seismic Line L1 reveals a thin (< 5 m) remnant deposit at the base of a slope (1°) separating surface T4 from surface T3 (Fig. 5a). This small lens of sediment appears to occupy a minor depression within surface T4 and oblique downlapping reflectors (sf_4_) indicate downslope progradation (Fig. 5c). Full recovery of vibrocore L1a revealed a very poorly sorted and poorly stratified succession of sandy mud, muddy sand and muddy gravel (Fig. 11). The gravel component comprises fine- to cobble-sized clasts of angular flint and chalk. Coarse grained beds are interbedded with finer grained beds dominated by a clay component. At the base of the core, highly weathered chalk confirmed that bedrock was reached. This deposit is interpreted as a mass wasting deposit at the base of a slope formed under cold climate conditions (periglacial colluvium; termed ‘Head’ regionally).

Preservation of sediments associated with the maximum erosion surface is rare and limited to a single palaeovalley fill (SB palaeovalley, Fig. 9a), as well as isolated deposits within the Northern Palaeovalley. The extent of these deposits is highlighted in Fig. 7b and the seismic stratigraphy presented in Figs. 5 and 6.

Sediments within the Northern Palaeovalley are limited to the margins of the main channel (Fig. 5a). The characteristic geometry, seismic stratigraphy and lithology of these deposits are shown on Figs. 5b and 11. Sediments partially fill the channel, are thickest at the channel margins, and thin towards the centre of the channel and outer channel banks. Seismic reflector patterns (sf_4_) indicate accretion towards the channel thalweg. Recovery of vibrocores was low due to the coarse clastic nature of sediments. Maximum recovery was achieved with N4c and N4d (Fig. 11) revealing matrix-supported gravel (Gm) interbedded with laminated silty fine sand (Sfp). Both the geometry of seismic facies and lithology of sediments imply deposition of a point bar within a bedrock fluvial channel. In the example shown in Fig. 5b at least three phases of accretion can be identified by separation of inclined reflectors by high amplitude reflectors. Within the channel thalweg, a bounding surface delimits the extent of accretion and slightly inclined to parallel onlapping reflectors mark a change in sedimentary regime (sf_2_, sf_3_ and sf_5_) with deposition of finer grained, less variable sediments.

The SB palaeovalley has a clear bathymetric and seismic expression and can be traced for 45 km in the eastern English Channel (Fig. 6a). Analysis of sub-bottom seismic profiles revealed a bedrock-confined single channel incised into deposits associated with T3 and infilled with up to 25 m of sediments (Fig. 6c). At the base of the channel is a thin (< 1 m) drape of sediment (sf_10_) that is succeeded by multi-storey stacking of sf_6_ (Table 1 and Fig. 6c). A vibrocore survey by Wessex Archaeology (2008) uncovered the lithology of deposits associated with each seismic facies, which is summarised in Table 3 and Fig. 6c. When tied to seismic facies, the sedimentary facies demonstrate a transition from coarse grained gravel (Gm) at the base of the channel to laminated sands (Sfp) within the lower channel-fill with increasingly shell rich sands towards the surface (Sh and Smw) (Table 2 and Fig. 6b). The association of seismic facies and sedimentary facies are interpreted to represent deposition of a coarse-grained basal lag subsequent to channel incision followed by deposition of estuarine and shallow marine sediments in response to sea-level transgression (Fig. 6c).

### 4.3 Stratigraphy and chronology

Erosion surfaces are cut into bedrock and overlain by shallow marine, coastal and fluvial deposits. Published OSL and ^14^C ages in the range of 176.6 ± 20 ka to 5.3 ± 0.5 ka (Table 3) provide a chronological constraint to deposition on the continental shelf over at least two glacial–interglacial cycles (Wessex Archaeology, 2008; Mellett et al., 2012b). OSL and ^14^C ages obtained by Wessex Archaeology (2008) are inconsistent within the same core, at the same depth, with a ^14^C age (9160–8350 Cal. BC) underestimating an OSL age (14.16 ± 1.1 ka) by approximately 3 ka (~ 20%).

**Fig. 12:**
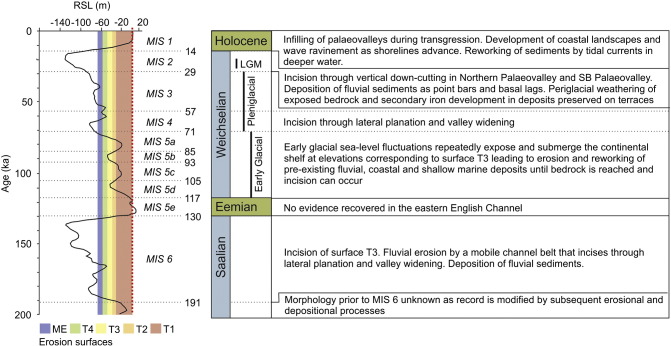
Erosional and depositional records preserved on the eastern English Channel continental shelf presented alongside the marine isotope record (Lisiecki and Raymo, 2005) and a composite global sea-level curve (Waelbroeck et al., 2002) during the mid- to late Quaternary. Chronostratigraphic subdivision is based on Cohen and Gibbard (2011). Timing of the Last Glacial Maximum (LGM) after Clark et al. (2009). Elevations of erosion surfaces shown on the sea-level curve with present-day sea-level highlighted by a thick dashed line. Interpretations of sedimentary regimes during each stage are based on the data presented in Section 4.

According to the chronometric data, sediments within the channel belt overlying surface T3 were deposited during marine oxygen isotope stage MIS 6 and the late stages of MIS 5 (Fig. 12). Exposure of surface T4 and deposition of sediments associated with surface ME occurred during MIS 2. This places formation of surface T4 at some point between the late sub-stages of MIS 5 and MIS 2. Partial infilling of surface ME and deposition of coastal sediments on surface T1 correlate to MIS 1 (Mellett et al., 2012b).

### 4.4 Formation of erosion surfaces

The similar morphologies of surfaces T1 and T2 (see Section 4.1) may suggest that they were formed by similar processes. Widespread erosion and formation of seaward-dipping planar surfaces can occur during sea-level transgression (Bradley and Griggs, 1976). Initial formation of surface T1 has been linked to marine planation during Neogene transgressions when the English Channel basin first became a marine embayment (Lautridou et al., 1986; Gibbard et al., 1988; Curry, 1989; Stride, 1990; Hamblin et al., 1992). The stepped margin separating surface T1 from all other surfaces has been interpreted as a submerged cliff line that formed during Neogene sea-level stillstands (Kellaway et al., 1975; Hamblin et al., 1992). The data presented here support such a model.

Using formation of T1 as a basis, surface T2 can also be considered the product marine planation, but during a later phase of marine transgression. Erosion of this surface would have occurred prior to opening of the Straits of Dover, dated to MIS 12 (Toucanne et al., 2009a). Surface T2 is drained by a dendritic channel network. Given the limited size of these channels it is unlikely that they acted as a conduit for drainage during breaching of the Straits of Dover and probably reflect more localised incision in response to regression. Sediments currently preserved on surfaces T1 and T2 originate from palaeovalley infill and shoreline retreat during the Holocene transgression (Table 3). Therefore, a significant hiatus between erosion and deposition is apparent.

Seismic profiles indicate that surface T3 formed through fluvial erosion by individual channels within a channel belt complex that combined to create a bedrock strath surface. Individual channels show evidence of lateral and downstream mobility that together with transport of a coarse-grained bedload, represented by a basal lag, is responsible for incision and valley widening during cold stages (Lewin and Gibbard, 2010). A single OSL age of 176.6 ± 20 ka (Table 3) from fluvial deposits overlying surface T3 suggests that, at least locally, incision occurred during or prior to the early parts of MIS 6. However, individual palaeovalleys are infilled with transgressive deposits dating to the early sub-stages of MIS 5, which have subsequently been partially eroded and reworked by renewed phases of fluvial incision. Therefore, erosion of surface T3 cannot be linked to a single climatic event. Whilst surface T3 demonstrates characteristics of a strath surface, the variable lithological composition of sediments within the channel belt complex and the degree of variability recorded in seismic profiles suggests that composite erosion was achieved through repeated phases of incision in response to fluctuating sea levels, rather than channel avulsion across a broad fluvial plain.

Sediments preserved on surface T4 show that after incision the area was sub-aerially exposed and subjected to periglacial conditions during MIS 2. A hiatus between incision and deposition is inferred as localised weathering of bedrock alone cannot explain the formation of surface T4. Subsequent modification by surface ME precludes confident interpretation of the erosion mechanisms. Surface T4 may have encompassed the area now occupied by the Northern Palaeovalley, as remnants of this surface are preserved at the valley margins. Incision of parallel-aligned, linear grooves (low sinuosity channels in Fig. 9) show a morphological resemblance to bedrock furrows described in Richardson and Carling (2005). These features are typically attributed to fluvial erosion but they can also occur in tidal environments where bedrock is sufficiently erodible (Carling et al., 2009). In the eastern English Channel, these furrows/low sinuosity channels are confined to the relatively softer bedrock of the Hampshire–Dieppe Basin suggesting that underlying lithology has some control over their formation (Fig. 9b). Based on the available data, distinguishing between fluvial or tidal erosion is not possible. Elsewhere, surface T4 forms the base of a broad palaeovalley that is confluent with the Median Palaeovalley in the central English Channel (Fig. 1) and indicates that at least part of surface T4 formed through fluvial incision. Erosion of this surface occurred at some time during MIS 4–3 when the continental shelf was sub-aerially exposed (Fig. 12).

Planform and cross-sectional geometries of surface ME in the SB Palaeovalley suggest that incision was driven by downcutting of a bedrock-confined fluvial channel. Assuming deposition of a basal lag on top of surface ME is a product of the processes driving erosion, an OSL age from this deposit provides a chronometric constraint for incision at ca. 21 ka (Table 3).

A change in morphology between the SB Palaeovalley and the Northern Palaeovalley (Fig. 9a) indicates a change in erosive regime. Bedrock morphology of the Northern Palaeovalley has been interpreted as the product of high magnitude flow regimes linked to catastrophic flooding through the Straits of Dover (Smith, 1985; Gupta et al., 2007). In part this morphology is constructional resulting from deposition of sediments from a laterally migrating point bar along the margin of the Northern Palaeovalley (Fig. 5b). Fluvial regimes operated between 17 ka and 15.8 ka according to the deposits correlated to the oldest seismic facies (Table 3). Here, formation of the erosion surface is driven by fluvial processes as seismic reflectors are concordant with the underlying erosion surface (Fig. 5b).

The time relationship between the formation of mid-channel bedrock bars (Fig. 9a) and deposition of laterally migrating point bars in the Northern Palaeovalley (Fig. 5b) is unknown. The mid-channel bedrock bars may be a remnant of antecedent fluvial regimes or of megaflood processes (Gupta et al., 2007) prior to occupation of the Northern Palaeovalley by a mobile channel during MIS 2. Alternatively, erosion of the mid-channel bars may have occurred at the same time as deposition at the valley margins. Large meltwater-fed, cold-climate gravel-bed rivers can exhibit a full range of fluvial styles within the same channel reach (Vandenberghe, 2001) and alluvial point bars and bedrock mid-channel bars can be created simultaneously, especially in areas where bedrock is exposed and susceptible to periglacial weathering.

Secondary drainage networks of considerably smaller magnitude are located on the bedrock bar surfaces within the Northern Palaeovalley (Gupta et al., 2007), which have been interpreted as representing periods of ‘normal fluvial processes’. According to the data presented here, the secondary drainage networks identified by Gupta et al. (2007) are considerably undersized in comparison. These secondary drainage networks show a similar morphology to cross-bar channels commonly observed in gravel-bed braided rivers (Bridge and Lunt, 2006). Therefore an alternative hypothesis may suggest that these superimposed minor drainage networks are the result of cross-bar bedrock scour during periods of high flow.

Separation of seismic facies by an erosional surface within sediments preserved at the margin of the Northern Palaeovalley (Fig. 5b) marks a change in sedimentary regime after ca. 16 ka that is probably related to reworking by tides during Holocene sea-level rise. This shows the erosive power of tides propagating through and confined within the Northern Palaeovalley, as they can produce erosional bedforms characteristic of high magnitude flows, particularly in less resistant lithologies (Carling et al., 2009). Whilst it is not possible to fully resolve the nature of processes responsible for formation of the Northern Palaeovalley, it is apparent that incision is at least partly controlled by fluvial processes operating during MIS 2 and erosion by coastal and shallow marine processes during Holocene transgression.

A summary of the sedimentary processes driving evolution of the continental shelf evolution from MIS 6 to MIS 1, as interpreted from erosional and depositional evidence preserved in the eastern English Channel, is presented in Fig. 12.

## 5 Discussion

**Fig. 13:**
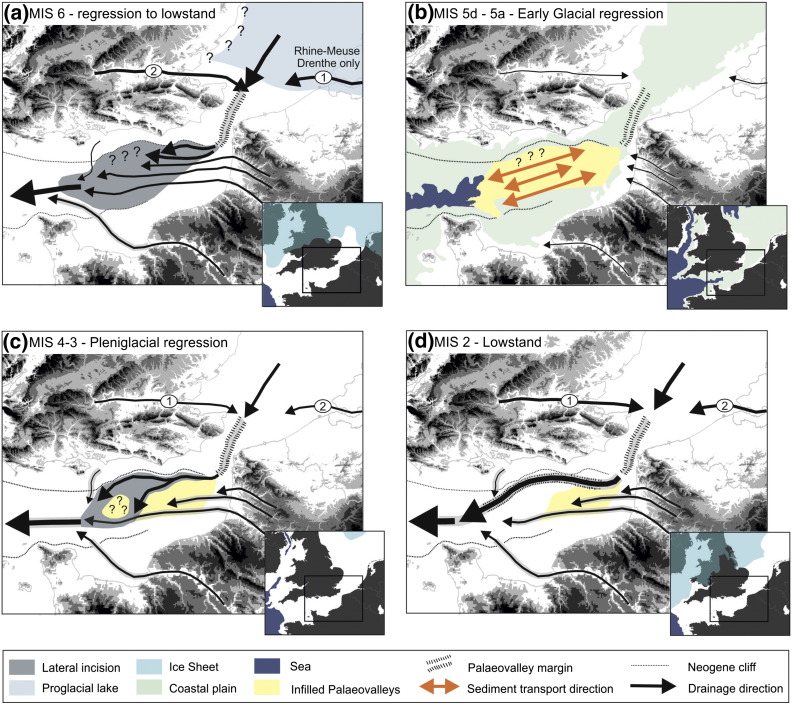
Palaeogeography and drainage configurations in the eastern English channel during; (a) MIS 6; (b) MIS 5d–5a; (c) MIS 4–3, and: (d) MIS 2. Black arrows show the direction of drainage and the thickness is proportional to discharge with thicker lines indicating higher discharges. The extent of the MIS 6 proglacial lake is based on Busschers et al. (2008) and Murton and Murton (2012). The position of the Rhine–Meuse (1) is taken from Busschers et al. (2008) and the position of the Thames (2) from Bridgland and D'Olier (1995). Ice margins are taken from Ehlers and Gibbard (2004). Shoreline locations are based on global relative sea-level history (Waelbroeck et al., 2002) and the bathymetry of the continental shelf.

Seismic stratigraphic, sedimentological and chronometric data from the eastern English Channel reveal that sculpting of the continental shelf occurred during multiple erosional phases resulting in the formation of a major composite erosion surface. Preservation of sediment on this bedrock erosion surface is typically confined to palaeovalleys or relict coastal landscapes. The data presented in this paper provide a framework to reconstruct the Middle to Late Pleistocene palaeogeographic configuration in the eastern English Channel (Fig. 13). Reconstructions are subdivided into time periods that correlate to the marine isotope record and Northwest European chronostratigraphic stages (Cohen and Gibbard, 2011). With the exception of the Holocene (MIS 1), interglacial periods are not discussed as no sedimentary records corresponding to these time periods were encountered in the eastern English Channel. This may have been due to the distribution of cores, where a bias towards coarser grained sediments was incorporated into the selection of sites. However, on non-subsiding continental shelves, interglacials are periods of relative sedimentary quiescence when compared to glacial stages, thus having less persistence in the sedimentary record (Hjelstuen et al., 2012). Further, there is a greater chance of reworking as successive erosion phases modify the morphological and sedimentary evidence of previous periods, and as a consequence the most recent phase of erosion and deposition is best preserved. The degree to which climate, relative sea level, sediment supply and discharge have influenced the timing and nature of shelf erosion and deposition is addressed by linking the submerged palaeovalleys of the eastern English Channel with pre-existing fluvial archives and sedimentary records at the shelf margin. Here we illustrate that knowledge of the timing and duration of processes operating at the basin-wide scale, and the persistence of landscapes thus created (cf. Brunsden, 1993), is essential to understand continental shelf evolution and distinguish between ‘normal’ and ‘catastrophic’ processes.

### 5.1 Palaeogeography and drainage configuration

#### 5.1.1 Saalian Drenthe (MIS 6)

Locally, fluvial incision of surface T3 corresponds to a period of climatic deterioration associated with the extensive Saalian (Drenthe substage) glaciation in Northwest Europe during MIS 6 (Graham et al., 2011). A significant base level fall of 120 m (Waelbroeck et al., 2002) would have steepened the fluvial profile and led to incision. Coalescence of the British and Fennoscandian ice sheets in the central North Sea Basin forced the Thames and Scheldt fluvial systems southwards towards the Straits of Dover (Bridgland et al., 1993; Bridgland and D'Olier, 1995; Gibbard, 1995; Bridgland and Gibbard, 1997). Incision at the Straits of Dover produced a lowered local base level which is reflected by widespread erosion in the Netherlands and southern North Sea (Busschers et al., 2008; Hijma and Cohen, 2011). This incision may have been driven or reinforced by potential ‘catastrophic’ discharge from a proglacial lake in the southern North Sea (Murton and Murton, 2012). However, the imprint such discharges leave on the landscape is difficult to determine and may be buffered by ‘normal’ background levels that are influenced by the dynamics of major NW European ice sheets and a very large catchment size. It is expected that effective drainage capture by the eastern English Channel of the Thames and Rhine–Meuse fluvial systems through the Straits of Dover would have increased sediment and water discharge above the capacity of existing rivers (Somme, Canche and Authie) and encouraged incision through the creation of new channel networks. Sediment supply would have been maintained by erosion of Mesozoic and Cenozoic strata, particularly Cretaceous chalk at the Straits of Dover, producing an abundant supply of flint for bedload transport within the palaeovalley network. During this time the fluvial regime would have been moderated by weathering and weakening of the underlying bedrock (Murton and Lautridou, 2003) and sediment flux derived from hillslopes (Murton and Belshaw, 2011), both conditioned by periglacial processes.

Seismic and bathymetric data show that the resulting palaeovalley network adopted a general E–W drainage configuration confluent with the Median Palaeovalley and Palaeo-Seine in the central English Channel (Lericolais et al., 2003) (Fig. 13a). The morphology preceding this erosion phase is unknown because parts of surface T3 form the oldest fluvial terrace preserved in the eastern English Channel. Incision may have extended northwards to the area that is now the Northern Palaeovalley and was potentially constrained by the morphology of surfaces T1 and T2. However, subsequent erosion of the record makes it difficult to confirm this. It is apparent that flow was confined to a single channel (Lobourg Channel, Fig. 1) in the relative uplands of the Weald–Artois anticline and became more distributive within the Hampshire–Dieppe Basin. This drainage configuration may simply have been the product of preceding morphology. However, there is potential for vertical incision and formation of the Lobourg Channel to have been enhanced by uplift and tilting of the Weald–Artois anticline in response to glacio-isostatic uplift in front of ice sheet margins (Busschers et al., 2008).

A single OSL age is available to constrain the incision to a period of increased ‘Channel River’ activity where peak discharges occurred at ca. 155 ka (Toucanne et al., 2009b). During this time discharge was primarily attributed to meltwater flux during ice sheet retreat between the Drenthe and Warthe advances of the Saalian glacial (Toucanne et al., 2009b), although Meinsen et al. (2011) suggested that catastrophic discharge from proglacial lakes in Northwest Europe may have flowed through the English Channel during this time.

#### 5.1.2 Early Weichselian glacial (MIS 5d–5a)

Relative sea-level fall during the transition from the Eemian (MIS 5e) to early Weichselian was not linear and globally sea-level fluctuated between a minimum of − 30 m and a maximum of − 80 m during MIS 5d–5a (Waelbroeck et al., 2002). This meant that for a long period (at least 55 ka) the eastern English Channel was a marginal marine environment where the interactions between fluvial and marine processes were controlled by multiple minor sea-level oscillations (Fig. 12). Fluvial systems during the initial sea-level fall of MIS 5d extended offshore onto the continental shelf. Most likely they reoccupied and modified valley networks formed during previous lowstands, and in many places, eroded sediments preserved within the palaeovalleys. As base-level continued to fall, incision of the underlying bedrock (surface T3) was initiated. During this time the palaeovalleys were most likely a conduit for sediment throughput and deposition appears to have been restricted to basal lags. Locally, lateral and downstream channel accretion has helped to preserve some sediments associated with earlier phases of deposition.

Minor transgressions punctuate the overall falling trajectory of sea level during the early glacial (Waelbroeck et al., 2002). At elevations where these transgressions flood palaeovalleys, there is the potential for deposition of coastal and shallow marine sediments as accommodation is created and shorelines step landward. Sediment supply may have been maintained through reworking of pre-existing deposits in a coastal plain setting as the interface between fluvial and marine processes shifted in response to changes in sea level (Fig. 13b). Phases of sediment reworking, incision and deposition as discussed above are expected to have operated over each stadial to inter-stadial sea-level cycle resulting in formation of a composite sheet of sediment and partial erosion of surface T3. There are no other deposits of the extent and thickness of the early Weichselian deposits preserved in the eastern English Channel. This reflects the uniqueness of the sea-level and climate history at these elevations during this period.

#### 5.1.3 Weichselian Pleniglacial (MIS 4–3)

Initial climatic deterioration and associated sea-level fall during late MIS 4 and MIS 3 led to sub-aerial exposure of the continental shelf in the eastern English Channel. Sediments deposited on surface T3 during MIS 6 to early sub-stages of MIS 5 were spared from significant physical reworking but subjected to chemical weathering, evident from the development of secondary iron, particularly in coarser grained sediments. During this time, ice sheets were expanding from Northern Scotland and Scandinavia (Ehlers and Gibbard, 2004) and the Thames and Rhine–Meuse fluvial systems occupied preceding channel networks and flowed south through the Straits of Dover into the eastern English Channel (Bridgland et al., 1993; Bridgland and D'Olier, 1995; Busschers et al., 2007). Sediment flux, as a proxy for discharge, through the ‘Channel River’ to the continental shelf margin was significantly less than during MIS 6 or MIS 2 (Toucanne et al., 2009b). Despite this, the eastern English Channel records an episode of substantial fluvial incision, suggesting discharge was high enough to enable erosion.

Extension of the Lobourg Channel into the eastern English Channel partially eroded sediments associated with earlier phases of palaeogeographic development (MIS 6–MIS 5) (Fig. 13c). Incision in the area of the Northern Palaeovalley occurred, potentially taking advantage of existing drainage configurations forged during previous sea-level lowstands. Valleys appear to have adopted a general E–W to NE–SW trend extending westwards to the continental shelf margin (Fig. 13c). The exact timing and nature of processes driving incision is unknown. Upstream, the Rhine–Meuse system records a phase of extensive reworking and lateral erosion during MIS 4 followed by a phase of aggradation during MIS 3 (Busschers et al., 2007). However, linking the timing of incision in the English Channel to the behaviour of the Rhine–Meuse system is problematic due to different time delays in the response to external controls along the catchment profile.

#### 5.1.4 Weichselian–Last Glacial Maximum (MIS 2)

Chronometric data for the SB Palaeovalley and Northern Palaeovalley places bedrock incision by fluvial processes in the period of maximum sea-level lowstand (− 120 m) at the Last Glacial Maximum (LGM) between 26.5 ka and 19 ka (Clark et al., 2009). During this time drainage beyond the Straits of Dover appears to have adopted a westerly direction following a structural fold (SB Palaeovalley) after emerging from the Lobourg Channel (Fig. 13d). The morphology of erosion surface ME suggests dominant flow then returned to a SW direction within the Northern Palaeovalley to become confluent with the Median Palaeovalley and Palaeo-Seine within the central English Channel (Fig. 1).

Given the relative timing during a sea-level lowstand period, the deep fluvial incision is interpreted to have been driven by steepening of the fluvial profile to a base-level that was maintained for a period of time sufficient to enable knickpoint retreat to extend 500 km upstream. Assuming that the knickpoint started at a lowstand shoreline where a noticeable change in the shelf gradient was exposed (0.00008 to 0.0002 at − 70 m OD), the rate of knickpoint retreat during the last glacial would be ca. 20 km/ka according to the duration of time that sea level was below this break in slope (Siddall et al., 2003), and the distance between the retreating shoreline and the knickpoint. The timing of incision and corresponding deposition within the Northern Palaeovalley correlates with a period of enhanced discharge through the English Channel that appears to be coupled to the collapse of the British and Fennoscandian ice sheets between 20 ka and 17 ka (e.g. Toucanne et al., 2010). Whilst it is expected that incision would principally be driven by relative sea-level fall, the depth of incision may have been enhanced or modified by sediment-laden waters at the onset of ice sheet collapse, the routing pattern guided by lithological changes and tectonic structures in the bedrock geology, and the rate of knickpoint retreat according to shelf topography. In addition, significant weakening of exposed bedrock at this time (surface T4) produced a basement susceptible to erosion.

Age-data place deposition within the Northern Palaeovalley between ca. 17 ka and 16 ka. In the Rhine–Meuse system, this time period is characterised by downstream sediment transport and reworking of older deposits (Busschers et al., 2007). Mineralogical analyses of sediments preserved within the Northern Palaeovalley indicate a partial Rhine origin (Busschers, pers. comm.). This would suggest that sediment routing from source to sink is not one of continuous throughput and bypass. The data presented here reveal that palaeovalleys on the continental shelf in the eastern English Channel act as transient or minor sediment sinks. However, a more highly resolved chronology is required to understand response and lag times along the catchment profile.

#### 5.1.5 Holocene (MIS 1)

In the eastern English Channel part of the palaeovalley network created during MIS 2 is infilled with deposits typically associated with a transgressive system tract (Miall, 2006). The sedimentary succession is comparable to palaeovalley fills preserved elsewhere in the English Channel during the Holocene sea-level transgression (Bellamy, 1995; Velegrakis et al., 1999; Gupta et al., 2004; Tessier et al., 2010). Deposition during relative sea-level rise is principally driven by the interaction between the creation of accommodation and sediment supply (Posamentier and Allen, 1999).

The SB Palaeovalley becomes confluent with the Northern Palaeovalley in the eastern English Channel. Whilst the SB Palaeovalley is completely filled, the Northern Palaeovalley is underfilled. This could be attributed to differences in valley morphology, where the Northern Palaeovalley has a greater cross-sectional area. Under these conditions an additional sediment source would be required to ensure the complete filling of the Northern Palaeovalley. However, the degree to which the Northern Palaeovalley was initially filled during early transgression is difficult to constrain, as due to the valley morphology there is significant potential for coastal and shallow marine processes during Holocene sea-level rise to rework any pre-existing valley infill. A bedload segregation zone in the Northern Palaeovalley around a tidal amphidromic point in the vicinity of the Isle of Wight triggered the transport of sediment towards the Straits of Dover (Anthony, 2002) and may have promoted excavation of the (partially filled) Northern Palaeovalley. The ability for tidal processes to entrain and erode would strongly depend on the sea bed morphology and grain size of the palaeovalley fill at different relative sea levels. Changes in the tidal regime in relation to reconnection of the North Atlantic with North Sea waters through the Straits of Dover at 8 ka (Scourse and Austin, 1995; Shennan et al., 2000) would complicate the matter further.

Deposits associated with sea-level transgression are not limited to palaeovalley fills (Mellett et al., 2012a). Coastal environments migrate landward in response to rising sea levels and, under certain circumstances, can be preserved. Reworking of finer sandy sediments into bedforms (sediment waves) and modification of sea bed morphology by tidal currents during the later stages of the Holocene is expected (Anthony, 2002; Cazenave, 2007).

### 5.2 Catastrophic flooding in the English Channel

It has been proposed that one or more catastrophic flood events where responsible for producing the erosive morphology of the Northern Palaeovalley in the English Channel (Smith, 1985; Gupta et al., 2007). The timing of this flood, or floods, has been linked to glacial-maxima during MIS 12 (Toucanne et al., 2009a) and/or MIS 6 (Meijer and Preece, 1995), and largely depends on the drainage of a proglacial lake in the southern North Sea Basin (Gibbard et al., 1988). The depositional record in the eastern English Channel is biased towards the most recent glacial–interglacial cycle and the sediments preserved are associated with relict coastlines and palaeovalley fills. No evidence was uncovered of constructional bedforms typically associated with catastrophic floods (e.g. Carling et al., 2009). However, the data presented here demonstrates that continental shelves are highly susceptible to reworking at the glacial–interglacial timescale. Therefore, if flood events did occur during MIS 12 and/or MIS 6, the sedimentary record of these events is likely to have been destroyed.

The erosional record in the eastern English Channel is composite and the morphology prior to MIS 6, particularly in the Northern Palaeovalley, cannot be constrained. There is evidence to suggest that ‘normal’ fluvial processes were at least partly responsible for shaping the Northern Palaeovalley during MIS 2, thus contradicting the conclusion by Gupta et al. (2007) that the morphology is the product of two discrete flood events. There is a possibility that the Northern Palaeovalley was sculpted by ‘catastrophic’ floods and then later reoccupied and modified by a fluvial system. However, to preserve the antecedent flood morphology would require the fluvial regime operating during subsequent sea-level lowstands to have limited erosive capabilities, which contradicts present models of fluvial behaviour in response to climate and sea-level change (Blum and Törnqvist, 2000; Vandenberghe, 2008; Gibbard and Lewin, 2009). As an alternative hypothesis it is suggested that multiphase fluvial processes operating over a long period of time (at least 200 ka) sculpted the eastern English Channel continental shelf, creating a landscape that has a comparable morphological character to one produced instantaneously by a catastrophic event, e.g. the Channeled Scablands (Bretz, 1969).

## 6 Conclusions

The landscape preserved on the present-day sea bed of the eastern English Channel is a record of the sedimentary processes that operated over glacial–interglacial sea-level cycles from MIS 6 to MIS 1. The mid- to late Quaternary landscape is a palimpsest of composite erosion surfaces formed through multiple phases of fluvial incision and a fragmentary and highly reworked depositional record that is biased towards sedimentary processes operating in shallow marine and coastal environments. The extensive volumes of coarse clastic sediments that are uncharacteristic for the erosional landscape of the English Channel continental shelf, and an essential aggregate resource, formed over multiple sea-level cycles as a multi-lateral, multi-storey succession of palaeovalley fills.

Fluvial incision occurs during relative sea-level fall or lowstand and correlates with periods of cold climatic conditions where bedrock is weakened by periglacial weathering. In part, fluvial incision may be coupled to ice sheet dynamics and ice front palaeogeography with increased discharges during phases of retreat. Palaeovalleys then infill as accommodation is created during sea-level transgression and sediment is supplied through bedrock and regolith erosion, and maintained through continuous recycling of terrestrial deposits over glacial–interglacial sea-level cycles. Evidence of sea-level highstands in the form of deposits during interglacial periods appears not to be preserved on the continental shelf. Further, the stratigraphic record is incomplete and sediments, as well as erosion surfaces, associated with each sea-level stage are only partially preserved. Over successive relative sea-level cycles the morphological and sedimentological expressions of sedimentary processes become superimposed and environmental information from the most recent glacial to inter-glacial cycle is preferentially preserved.

In the eastern English Channel there are no unequivocal erosional or sedimentological records of the processes operating during MIS 12 preserved. Understanding palaeogeographic development during this time, particularly in relation to the mechanisms driving breaching of the Weald–Artois anticline at the Straits of Dover, is problematic due to later bedrock erosion and multi-phase recycling of sediments. Evidence is preserved to support major erosion during MIS 6. However, sedimentary processes during this time alone cannot account for the morphology of the Northern Palaeovalley as it was at least partly re-sculpted by fluvial processes during MIS 2. Events of catastrophic magnitude are not necessarily responsible for formation of the palaeovalley network preserved in the eastern English Channel and it is much more likely that the landforms were created by processes of non-catastrophic magnitude operating over long (10^4^ yr) periods of time.

## Figures and Tables

**Table 1:** Seismic facies characteristics. Refer to Fig. 3 for a guide to the interpretation of reflector configurations and facies geometries.

Seismic facies	Internal geometry	Frequency	Amplitude	Continuity	Reflector configuration	Reflector termination
sf_1_	Sheet	Medium to high	High	Continuous	Parallel to low angle parallel oblique	Onlap
sf_2_	Channel	Medium to low	High	Continuous	Parallel draped	Concordance
sf_3_	Channel or lens	medium	High	Continuous to discontinuous	Sub-parallel	Onlap
sf_4_	Channel or lens	Medium to low	Medium to high	Continuous to discontinuous	Oblique to sigmoid	Downlap and truncation
sf_5_	Lens	Medium	Medium to high	Continuous	Parallel oblique	Downlap and truncation
sf_6_	Channel	Medium to high	medium	Discontinuous	Oblique to hummocky	Downlap and truncation
sf_7_	Channel	Medium	Medium to low	Discontinuous	Hummocky to chaotic	–
sf_8_	Channel	Medium to low	Low	Discontinuous	Undifferentiated	–
sf_9_	Mound	Medium	Medium to high	Discontinuous	Oblique to sigmoid	Downlap and truncation
sf_10_	Drape	Reflectors below resolution of unit				

**Table 2:** Classification and interpretation of sedimentary facies according to lithological composition.

Sedimentary facies	Description	Depositional environment/process
Gm	Very poorly sorted matrix supported sandy gravel. Gravel typically comprises sub-angular to sub-rounded flint clasts. Matrix is medium to coarse sand.	Fluvial
Gfu	Well sorted clast supported coarse gravel up to cobble size. Gravel is largely flint and sub-rounded	Coastal (nearshore)
Sfp	Fine to medium sand with frequent laminations of silty clay. Laminations are generally fine but can be up to 1 cm in thickness. Clay is occasionally organic rich.	Alluvial or tidally influenced
Sh	Poorly sorted slightly gravelly fine to medium sand with frequent outsized gravel clasts. Gravel component is sub-angular to sub-rounded, fine to medium size and of various lithologies. Frequent shell fragments throughout.	Shallow marine to coastal (nearshore)
Smw	Generally well sorted occasionally moderately sorted slightly gravelly medium to coarse calcareous sand. Shells, both whole and fragmented are frequent. Occasional organic mottles and inclusions of granule size coal.	Coastal–shallow marine
Sfw	Very well sorted laminated fine to medium size quartz sand. Occasional inclusions of coal of granule size.	Shallow marine (shoreface)
Cd	Very poorly sorted silty sandy gravelly clay. Gravel is largely chalk and flint. Deposit is stiff and varies considerably in composition.	Periglacial slope
Cm	Very poorly sorted matrix supported gravel. Matrix is clayey sandy silt with frequent shell fragments throughout. Gravel is angular to sub-rounded, fine to medium size.	Wave/tide reworking
Br	Bedrock, often highly weathered.	–

**Table 3:** Age data. Locations of cores, with the exception of those recovered by Wessex Archaeology (2008), are shown in Fig. 2. Refer to cited literature for full methodological details. Uncertainty on OSL ages is given at 1-σ level and for ^14^C ages, at 2σ level. MAM-3—minimum age model with 3 parameters.

Erosion surface	Location	Depositional environment	Core	Depth (m OD)	Dating method	Age	Reference	Reliability
T1	Hastings Bank	Coastal (washover fan)	VC37a	− 16.81	OSL	5.3 ± 0.5 ka	Mellett et al. (2012a, 2012b)	Incomplete bleaching, MAM-3 applied
		Shallow marine	VC28	− 19.11	OSL	7.8 ± 0.2 ka		Well bleached
		Shallow marine	VCL3b	− 13.70	OSL	8.4 ± 0.2 ka		Well bleached
		Coastal (nearshore beach)	VC37b	− 24.02	OSL	8.0 ± 0.6		Incomplete bleaching, MAM-3 applied
T3	Channel belt complex	Shallow marine	VC52b	− 48.80	OSL	107.8 ± 5.2 ka	Mellett et al. (2012a) Wessex Archaeology (2008)	Well bleached
	SB palaeovalley (alluvial terrace)	Marine marginal	VC7	− 41.56	OSL	83.2 ± 6.6 ka		No information published
		Fluvial (palaeosol)		− 42.88	OSL	176.6 ± 20 ka		
T4	Northern Palaeovalley margin	Periglacial (head deposit)	VCL1a	− 53.10	OSL	17.9 ± 0.7 ka	Mellett et al. (2012b)	Potential post-depositional mixing and variable dose rate
ME	Northern Palaeovalley	Fluvial (point bar)	N4c	− 53.20	OSL	15.8 ± 0.9 ka	Mellett et al. (2012b)	Well bleached
		Fluvial (point bar)	N4d	− 53.40	OSL	16.8 ± 0.7 ka		
		Fluvial (point bar)	N4d	− 52.70	OSL	17.1 ± 0.8 ka		
	SB Palaeovalley	Shallow marine	VC1	− 47.90	C^14^ (shell)	7320–6860 cal. BC	Wessex Archaeology (2008)	Marine reservoir effect
		Marine marginal	VC1	− 48.03	OSL	11.9 ± 0.9 ka		No information published
			VC1	− 48.86	C^14^ (shell)	9160–8150 cal. BC		Marine reservoir effect
		Marine marginal (estuarine to freshwater)	VC3	− 55.12	OSL	14.2 ± 1.1 ka		No information published
			VC3	− 55.13	C^14^ (shell)	9160–8350 cal. BC		Marine reservoir effect
		Alluvial or tidally influenced	VC3	− 55.58	OSL	15.1 ± 1.2 ka		No information published
		Fluvial (basal lag)	VC5	− 49.34	OSL	21.2 ± 1.5 ka		No information published
